# Carbapenem-Resistant *Enterobacter cloacae* Isolates Producing KPC-3, North Dakota, USA

**DOI:** 10.3201/eid2009.140344

**Published:** 2014-09

**Authors:** Lee M. Kiedrowski, Dubert M. Guerrero, Federico Perez, Roberto A. Viau, Laura J. Rojas, Maria F. Mojica, Susan D. Rudin, Andrea M. Hujer, Steven H. Marshall, Robert A. Bonomo

**Affiliations:** North Dakota State University, Fargo, North Dakota, USA (L.M. Kiedrowski);; Sanford Health, Fargo (D.M. Guerrero);; Louis Stokes Cleveland Department of Veterans Affairs Medical Center, Cleveland, Ohio, USA (F. Perez, R.A. Viau, L.J. Rojas, M.F. Mojica, S.D. Rudin, A.M. Hujer, S.H. Marshall, R.A. Bonomo);; Case Western Reserve University School of Medicine, Cleveland (F. Perez, R.A. Viau, L.J. Rojas, M.F. Mojica, S.D. Rudin, A.M. Hujer, S.H. Marshall, R.A. Bonomo)

**Keywords:** outbreak, KPC, Enterobacter cloacae, Carbapenem resistance, antimicrobial resistance, bacteria, North Dakota, United States

**To the Editor:** Carbapenem-resistant *Enterobacteriaceae* (CRE) continue to emerge as a serious public health threat throughout the world (*1*). CRE infections in the United States are often mediated by acquisition of *Klebsiella pneumoniae* carbapenemase (KPC) expressed by *Klebsiella* spp., although KPC is also found in other genera (*2*). The spread of KPC-producing, gram-negative bacteria in hospitals has been linked to severity of illness, co-existing medical conditions, exposure to antimicrobial drugs, and need for chronic care (*3*).

After reporting of CRE infections to the North Dakota Department of Health became mandatory in 2011, a total of 20 CRE cases were noted in 12 of 53 counties (2.9 cases/100,000 population [*4*]). Most cases involved infection with *Enterobacter cloacae* and occurred in Cass County, where the state’s largest city, Fargo, is located. We describe an outbreak of clonal carbapenem-resistant *E. cloacae* in a health care system in Fargo.

Sanford Health is a 583-bed, acute-care facility, representing ≈70% of acute-care beds in Fargo. The hospital handles >27,000 admissions/year and serves as a referral center for a large area of the state, and the only long-term acute-care (LTAC) facility in the eastern half of the state operates on its campus. During December 2011–December 2012, all isolates of *Enterobacteriaceae* with reduced susceptibility to ertapenem (MIC ≥1 µg/mL) identified at the hospital’s clinical microbiology laboratory were screened for carbapenemase production by using the modified Hodge test (mHT), according to Clinical and Laboratory Standards Institute recommendations (*5*). Identification and susceptibility testing were done with the MicroScan system (Siemens Healthcare Diagnostics, Tarrytown, NY, USA); MICs of carbapenems were confirmed with Etest (bioMérieux, Durham, NC, USA). Three carbapenem-resistant *E. cloacae* isolates from documented cases of CRE infection at the hospital during 2010 were analyzed for comparison.

To characterize carbapenem-resistant and mHT-positive isolates, we used PCR to amplify and sequence the carbapenemase genes *bla*_IMP_, *bla*_NDM_, *bla*_VIM_, and *bla*_KPC_ by using established methods (*6*). The upstream sequence of *bla*_KPC_-positive strains was analyzed to determine the isoform of the transposon Tn*4401* that harbored *bla*_KPC_ (*7*). We investigated genetic similarity among isolates by repetitive sequence-based PCR; isolates with >95% similarity were considered clonal (*6*). We also sequenced the highly conserved *hsp60* gene (*8*) and attempted conjugative transfer of the *bla*_KPC_ gene by growing KPC-producing *E. cloacae* along with sodium azide–resistant *Escherichia coli* J-53. As part of the study, we examined records of patients from whom carbapenem-resistant *E. cloacae* was isolated. The study was approved by the Institutional Review Board at Sanford Health.

During December 2011–December 2012, a total of 19 single-patient *E. cloacae* isolates and 1 *E. aerogenes* isolate had positive mHT results. *bla*_KPC_ was detected in 17 of the 19 *E. cloacae* isolates and in the 3 carbapenem-resistant *E. cloacae* isolates from 2010. For all 20 of those isolates, sequencing revealed *bla*_KPC-3_ in association with isoform d of the transposon Tn*4401*, and all isolates were clonally related (Figure). All 20 isolates also had an identical *hsp60* sequence belonging to cluster VI in the Hoffman and Roggenkamp scheme (*8*). Conjugation of a *bla*_KPC_-containing plasmid into *E. coli* J-53 was successful for 1 strain. 

**Figure:**
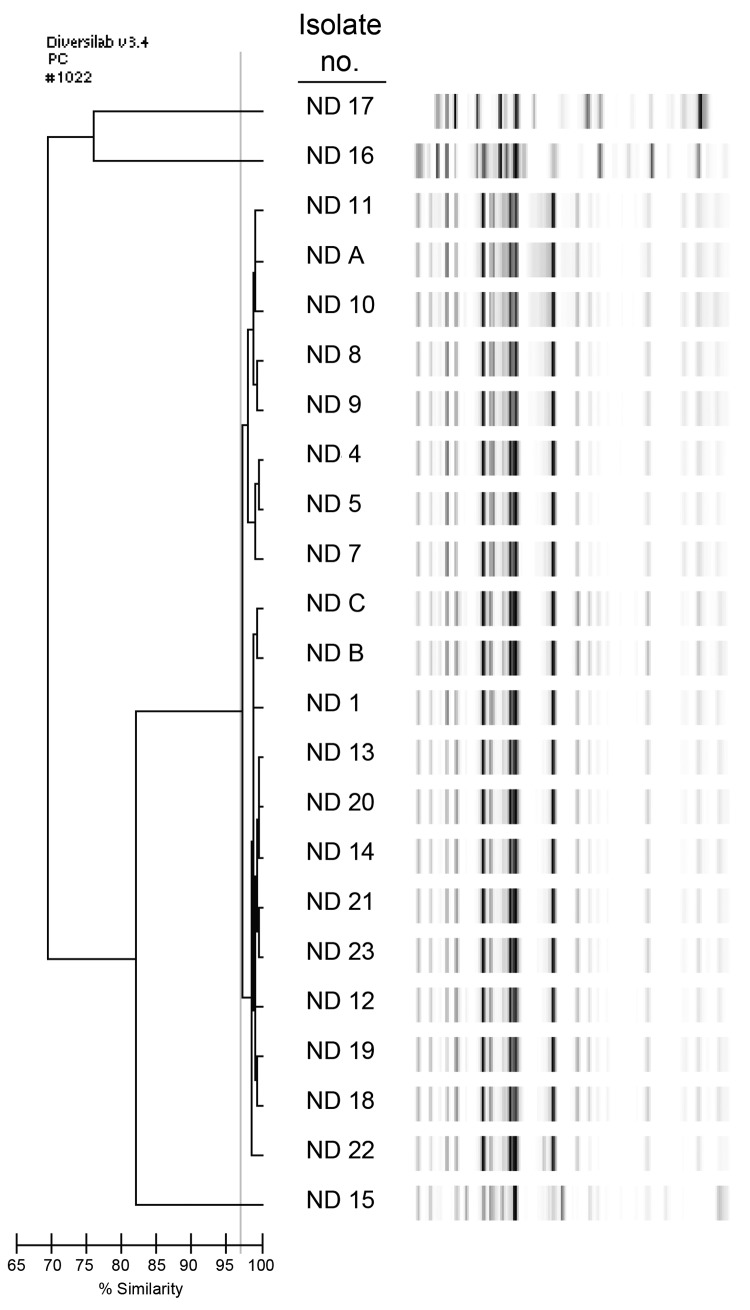
Genetic typing of carbapenem-resistant *Enterobacter cloacae* identified from patients at Sanford Health in Fargo, North Dakota, USA. Repetitive sequence–based PCR was used. The dendrogram at left displays the percentage similarity among band patterns shown at right. Isolate numbers ND 1, ND 4–5, ND 7–14, and ND 18–23 indicate *Klebsiella pneumoniae* carbapenemase (KPC) 3–producing *E. cloacae* isolates isolated during December 2011–December 2012; ND A–C indicate KPC-3–producing *E. cloacae* isolated during 2010. All KPC-3–producing *E. cloacae* isolates share >97% similarity, indicating a clonal strain. ND 15 and 16 are *E. cloacae*, and ND 17 is *E. aerogenes*, genetically distinct and without carbapenemases.

All 20 of the patients from whom KPC-producing CRE isolates were obtained (17 from this study, 3 from 2010) had been hospitalized at Sanford Health during the 3 months before CRE isolation; 13 (65%) were admitted to intensive care. In addition, 13 (65%) patients had been admitted to the LTAC during the year before CRE isolation. Co-colonization with multidrug-resistant bacteria was documented in 16 (80%) patients, including extended-spectrum β-lactamase–producing and carbapenem-resistant organisms in 4 and 2 patients, respectively. Seven (35%) patients died; 3 (15%) deaths were attributed to CRE infection. One of the patients was a neonate 30 days of age.

The finding of KPC-3–producing *E. cloacae* in North Dakota contrasts with the predominant epidemiology of CRE across the United States. Most CRE cases nationwide are caused by KPC-producing *K. pneumoniae* (*2*). KPC-type β-lactamases were previously identified in diverse strains of *Enterobacter* spp. from an urban health care system in Detroit, accounting for ≈15% of CRE (*9*). In contrast, our genetic analysis reveals a uniform genetic background among KPC-producing *E. cloacae*, which suggests horizontal dissemination of an outbreak strain.

Because active surveillance programs do not exist at our facility, this study probably underestimates the extent of CRE spread. We found that patients with KPC-producing *E. cloacae* in this sample were exposed to an LTAC and concomitantly were colonized or infected with other multidrug-resistant organisms (*9*). Although the spatio-temporal origin of the outbreak (acute care vs. LTAC) remains undefined, these findings likely reflect longer exposure to the continuum of care and higher rates of co-existing conditions within the LTAC population. This outbreak of KPC-producing *E. cloacae* infections in a health care system in North Dakota highlights the infection control challenges of long-term care facilities and the potential role they play in CRE dissemination.

## References

[1] Munoz-Price LS, Poirel L, Bonomo RA, Schwaber MJ, Daikos GL, Cormican M, Clinical epidemiology of the global expansion of *Klebsiella pneumoniae* carbapenemases. Lancet Infect Dis. 2013;13:785–96 . 10.1016/S1473-3099(13)70190-7 23969216 PMC4673667

[2] Kitchel B, Rasheed JK, Patel JB, Srinivasan A, Navon-Venezia S, Carmeli Y, Molecular epidemiology of KPC-producing *Klebsiella pneumoniae* isolates in the United States: clonal expansion of multilocus sequence type 258. Antimicrob Agents Chemother. 2009;53:3365–70 . 10.1128/AAC.00126-09 19506063 PMC2715580

[3] Swaminathan M, Sharma S, Poliansky Blash S, Patel G, Banach DB, Phillips M, Prevalence and risk factors for acquisition of carbapenem-resistant *Enterobacteriaceae* in the setting of endemicity. Infect Control Hosp Epidemiol. 2013;34:809–17. 10.1086/671270 23838221

[4] North Dakota Department of Health. Carbapenem-resistant. *Enterobacteriaceae* (CRE) including *Klebsiella pneumoniae* carbapenemase (KPC) producers [cited 2014 Jan 20]. http://www.ndhealth.gov/disease/cre

[5] Clinical and Laboratory Standards Institute. Performance standards for antimicrobial susceptibility testing. 19th informational supplement. Wayne (PA): The Institute; 2009.

[6] Perez F, Endimiani A, Ray AJ, Decker BK, Wallace CJ, Hujer KM, Carbapenem-resistant *Acinetobacter baumannii* and *Klebsiella pneumoniae* across a hospital system: impact of post-acute care facilities on dissemination. J Antimicrob Chemother. 2010;65:1807–18 . 10.1093/jac/dkq191 20513702 PMC2904665

[7] Naas T, Cuzon G, Villegas MV, Lartigue MF, Quinn JP, Nordmann P. Genetic structures at the origin of acquisition of the β-lactamase *bla*_KPC_ gene. Antimicrob Agents Chemother. 2008;52:1257–63. 10.1128/AAC.01451-07 18227185 PMC2292522

[8] Hoffmann H, Roggenkamp A. Population genetics of the nomenspecies *Enterobacter cloacae.* Appl Environ Microbiol. 2003;69:5306–18 . 10.1128/AEM.69.9.5306-5318.2003 12957918 PMC194928

[9] Marchaim D, Chopra T, Perez F, Hayakawa K, Lephart PR, Bheemreddy S, Outcomes and genetic relatedness of carbapenem-resistant *Enterobacteriaceae* at Detroit medical center. Infect Control Hosp Epidemiol. 2011;32:861–71 . 10.1086/661597 21828966 PMC4067763

